# Role of publicly funded health insurance in financial protection of the elderly from hospitalisation expenditure in India-findings from the longitudinal aging study

**DOI:** 10.1186/s12877-022-03266-2

**Published:** 2022-07-12

**Authors:** Samir Garg, Kirtti Kumar Bebarta, Narayan Tripathi

**Affiliations:** State Health Resource Centre, Chhattisgarh, Raipur India

**Keywords:** Elderly, Healthcare, Out of pocket expenditure, Insurance, Catastrophic expenditure, Financial Protection, Universal health coverage, India

## Abstract

**Background:**

The elderly face a greater burden of illnesses than other age groups and have a more frequent need of healthcare, including in-patient hospitalisations. Catastrophic expenditure on hospitalisation of the elderly poses a significant challenge to India’s aim of achieving Universal Health Coverage (UHC). India has implemented a policy of Publicly Funded Health Insurance (PFHI) to provide free inpatient care by empanelling private and public hospitals. The existing studies have examined the performance of PFHI in financial protection of the elderly.

**Methods:**

This study utilised the Longitudinal Ageing Study in India (LASI) Wave 1, conducted in 2017–18. LASI is a large-scale nationally representative survey collecting data on elderly health including illness burden, healthcare use and out of pocket expenditure (OOPE). It covered a sample 72,250 individuals aged 45 or above. Financial Protection was measured in terms of Catastrophic Health Expenditure (CHE). Multivariate analysis was conducted to find effect of PFHI on OOPE—quantile and logistic models were applied for OOPE and CHE respectively. For robustness, Propensity Score Matching (PSM) model was applied.

**Results:**

Of the hospitalisations, 35% had taken place in public hospitals. The mean OOPE for a hospitalisation in public sector was Indian Rupees (INR) 8276, whereas it was INR 49,700 in private facilities. Incidence of CHE was several times greater for using private hospitals as compared to public hospitals. Multi-variate analyses showed that enrolment under PFHI was not associated with lower OOPE or CHE. PSM model also confirmed that PFHI-enrolment had no effect on OOPE or CHE. Use of private facilities was a key determinant of OOPE, irrespective of enrolment under PFHI.

**Conclusions:**

This was the first study in India to examine the performance of PFHI in the context of catastrophic hospitalisation expenditure faced by the elderly. It found that PFHI was not effective in financial protection of the elderly. The ongoing reliance on a poorly regulated private sector seems to be a key limitation of PFHI policy. Governments need to find more effective ways of protecting the elderly from catastrophic health expenditure if the goal of UHC has to be realized.

**Supplementary Information:**

The online version contains supplementary material available at 10.1186/s12877-022-03266-2.

## Background

The share of the elderly in global population has been rising including in the Low and Low-to Middle-Income countries (LLMICs) [1–3]. The elderly face a greater burden of illnesses than other age groups and thereby have greater need for healthcare [4–6]. India has a large population of the elderly [7]. According to the last national census held in 2011, India had 104 million persons above the age of 60 years and they constituted 8.6% of its total population [3].

The recent studies in India show that a rapidly aging population is facing increasing burden of chronic health problems. While the non-communicable diseases have been rising everywhere, many parts of India continue to have a significant burden of communicable diseases also [8–12]. This situation means a more frequent need of healthcare for the elderly, including in-patient hospitalisations. The hospital care of the elderly is resulting in large out of pocket expenditure (OOPE) [8–12].

Nations across the world have adopted Universal Health Coverage (UHC) as the desired goal for health systems and ensuring protection from catastrophic health expenditures is central to it. Studies in India and other LLMICs have shown that hospitalisations of the elderly often lead to catastrophic expenditure for the elderly and their families [8, 13–18]. The occurrence of large OOPE on hospitalisations of the elderly poses a significant challenge to India’s aim of achieving UHC, [8, 11, 19].

Publicly Funded Health Insurance (PFHI) schemes are seen as important means to achieve UHC in LLMICs as a strategy for expanding access and ensuring financial protection for healthcare [20]. These demand-side financing mechanisms entitle poor and other vulnerable households to choose cashless healthcare from a pool of empanelled providers [21]. PFHI programmes have existed in India for more than a decade now [21]. PFHI in India has been aimed at providing free inpatient care by empanelling private and public hospitals [20, 21].

The elderly are an important group to be covered under PFHI so that they are protected from large out of pocket expenditure when they need to utilise healthcare. It is therefore crucial to know how PFHI has performed in achieving the above objective..Despite the important role of PFHI in financial protection, none of the existing studies in India have examined its performance in protecting the elderly from OOPE and catastrophic expenditure. The current study was therefore aimed at addressing the above gap in literature.

PFHI in India: PFHI schemes started in some states of India around 2006 and a national scheme got launched in 2008 [20, 21]. Thereafter most states in India have implemented PHI schemes to cover the poor households and other vulnerable populations. PFHI in India mainly covers inpatient care i.e. hospitalisation expenditure [20, 21]. Government carries out the enrolment of the eligible households and individuals. Some states engage insurance firms as intermediaries. Other state governments set up their own ‘Trusts’ to act as a purchaser organisations [20, 21]. The states or their purchaser organisations enter into contracts with private and public hospitals. Government announces a defined list of services covered under the scheme and the pre-defined prices at which hospitals will get reimbursed. Hospitals interested in joining the scheme apply for empanelment and those who pass the government's scrutiny for defined capacity and quality parameters enter into contracts [20, 21]. The contracted hospitals provide services to enrolled individuals and generate claims through an online process to get reimbursed at pre-defined prices. The services under PFHI are expected to be completely free for the enrolled persons and 'cash-less' at the point of care [20, 21]. The contracts with the hospitals prohibit them to charge any copayments from the patients. The contracts with hospitals are renewed annually [20, 21].

## Methods and materials

### Dataset

This study utilised the Longitudinal Ageing Study in India (LASI), Wave 1 that was conducted in 2017–18 [22]. LASI is planned to be conducted every 3 years for the next 25 years. LASI is a large-scale national survey collecting data on burden of disease, functional health, healthcare financing, utilisation, and the social and economic wellbeing of the elderly population.

LASI has a multi-stage sampling design with a nationally representative sample of rural and urban population of older adults (aged 45 and above). It covers all states of India. In its first wave, it covered a sample of 72,250 individuals [22]. The sample size was sufficient for the purpose of the current study.

It collected information on demographic details, household characteristics, food and non-food consumption expenditure; self reported morbidity, healthcare utilisation, household health expenditures (OOPE) and health insurance coverage.

### Data analysis

LASI survey data was analysed using STATA V.14. Sampling weights were applied to account for both study design (stratification) and non-response [22]. Individual response rate was 87.3%. Descriptive statistics was used to calculate mean and median OOPE with 95% confidence intervals (CI). Out of pocket expenditure (OOPE) for each episode included all medical expenses and the expenses on transportation.

Financial Protection was measured in terms of Catastrophic Health Expenditure (CHE) as proposed by Wagstaff and Doorslaer [23]. The survey collected data on consumption expenditure of households on food and non-food purposes. This study used two types of measures for CHE:

- CHE as a proportion of annual non-food consumption expenditure: A threshold of 40% of concerned household’s annual non-food consumption expenditure were taken for CHE and named CHE40. This is a commonly used measure of calculating CHE [23].
- CHE as a proportion of annual consumption expenditure: A threshold of 25% of concerned household’s total annual consumption expenditure were taken for CHE and named CHE25. Recent studies in India have used such a measure for CHE [20, 21, 24].

The list of the variables included in this study is given in Table1**.**

**Table 1:** List of variables included in the study

**Variables**	**Description**	**Category**
Place	Place of Residence of Individual	Urban
		Rural
MPCE Quintile	Monthly per capita consumption expenditure in INR and household non-food expenditure as a share	Poorest
		Poorer
		Middle
		Richer
		Richest
Social Group (Caste)	Social Group/Caste of Individual	Scheduled Tribe (ST)
		Scheduled Caste (SC)
		backward class (OBC)
		None of above
Age Category	Age Category of Individual	45–59 Years
		60–79 Years
		80 and Above
Sex	Sex of Individual	Male
		Female
Education	Education Category of Individual	Never been to school
		Primary
		Secondary
		Higher Secondary and Diploma
		Graduation and Above
PFHI	Public Funded Health Insurance	Yes
		No
Type of Hospital	Type of Hospital in which hospitalisation took place	Public Hospital
		Private Hospital
Duration of Hospitalisation (Days)	Duration of Hospitalisation Episode in no. of days	Continuous
OOPE	Out of Pocket Expenditure (OOPE) in hospital in INR	Continuous
CHE25	Catastrophic Health Expenditure at 25% threshold of annual consumption expenditure (OOPE > 25% of Annual Consumption Expenditure of concerned Household)	Yes
		No
CHE40	Catastrophic Health Expenditure at 40% threshold of Non-food consumption expenditure (OOPE > 40% of Annual Non-food Consumption Expenditure of concerned Household)	Yes
		No
Disease	Type of ailment for which hospitalisation took place	Names of diseases
State	State in which the individual resides	Names of states in India

Multivariate quantile regression analysis was carried out to examine the effect of PFHI on size of OOPE [25]. Quantile regression was used to address the possibility of any skew or extreme values in OOPE. For comparison of results, Ordinary Least Squares (OLS) regression was also carried out for OOPE and reported alongside. The independent variables included in the regression models were selected based on the existing studies of PFHI in India [20, 21, 24].

Multivariate logistic regression analysis was used to find out the association of enrolment under PFHI with CHE25 and CHE40.

For robustness, Propensity Score Matching (PSM) model was applied to examine the effect of enrolment under PFHI on OOPE, CHE25 and CHE40. PSM was meant to confirm whether OOPE differed significantly between episodes of the PFHI-enrolled individuals and the non-enrolled individuals while other relevant characteristics were matched. PSM has been recommended as a suitable method for making such comparisons because it can help in achieving a better balance in patient characteristics [26, 27]. The application of the PSM model includes computing the propensity scores and treatment effect. The average treatment effect on the treated (ATET) gets computed by taking the average of the difference between the observed and potential outcomes for each subject [28]. In the current analysis, the enrolment status of the individual (PFHI enrolled/non-enrolled) was used as the treatment variable while applying the PSM model in STATA. PSM has been used by many studies for evaluating PFHI schemes on financial protection [20, 21, 24, 29].

## Results

The sample profile of individuals covered under LASI wave 1 is given in Table 2.

**Table 2:** Sample profile

Variables	Values (N = 72,250)
**Place**	
Urban	35.61%
Rural	64.39%
**Household Size**	
Upto 5 members	63.80%
Above 5 Members	36.20%
**Monthly Per Capita Consumption Expenditure (MPCE) Quintile**	
Poorest	19.67%
Poor	20.10%
Middle	20.14%
Rich	20.26%
Richest	19.82%
**Caste (Social Group)**	
Scheduled Tribes (ST)	17.21%
Scheduled Castes (SC)	16.98%
Other Backward Classes (OBC)	37.76%
Others (None of the above)	28.05%
**Age**	
45–59 Years	56.45%
60–79 Years	38.86%
80 and Above	4.69%
**Sex**	
Male	42.31%
Female	57.69%
**Education**	
Never been to school	46.77%
Primary	24.18%
Secondary	19.02%
Higher Secondary and Diploma	4.89%
Graduation and Above	5.14%
**PFHI enrolment**	
Yes	18.13%
No	81.87%

Around two-third of the elderly were residents of rural areas. Around a third of the elderly belonged to the vulnerable social groups of the scheduled castes and scheduled tribes. Less than 5% of the elderly were above 80 years old and 56% were younger than 60 years. Around half the elderly did not have any formal education.

Overall, 18.13% of the elderly were covered under PFHI. The proportion of those covered under other kinds of health insurance was relatively small, at 2.58%.

### Hospitalisation rates among elderly under PFHI schemes

The overall hospitalisation rate was 7.1% (6.5%-7.6%) of the elderly (Table 3). Among the PFHI enrolled, the hospitalisation rate was 8.8% (8.2%-9.4%), whereas it was 6.8% (6.2%-7.5%) among those not enrolled under PFHI.

**Table 3:** Share of public and private facilities in hospitalisations of the elderly with 95% CI

Overall (*N* = 4783)	Public Facility (%)	Private Facility (%)
	***n* = 3,105**	***n* = 1633**
All	35.06 (31.8–38.5)	64.9 (61.5–68.2)
PFHI Enrolled	44.58 (39.4–49.9)	55.42 (50.1–60.6)
Not Enrolled	32.57 (28.9–36.4)	67.43 (63.5–71.1)

The overall share of public facilities in the hospitalisations of the elderly was 35.06% (31.8%-38.5%) and the rest was in private hospitals (Table 2). Public sector utilisation among the PFHI-enrolled individuals was greater than those not enrolled under PFHI (Table 2).

### Out of pocket expenditure (OOPE)

Overall, the mean OOPE for utilising hospitalisation care in private sector was around six times greater than in public sector (Table 4). A similar ratio was seen in mean OOPE incurred in private and public facilities by the PFHI-enrolled individuals.

**Table 4:** Mean and Median OOPE for per episode of hospitalisation (in INR) with 95% CI () in PFHI-enrolled and not-enrolled elderly

a. Mean OOPE for per episode of hospitalisation (in INR) with 95% CI			
(*N* = 4,616)	Mean OOPE (CI)	Public facilities	Private facilities
All	35,249 (32,813–37,684)	8276 (6625–9928)	49,700 (45,974–53,426)
PFHI Enrolled	18,650 (16,083–21,217)	5092 (4144–6039)	29,454 ( 24,946–33,963)
Not enrolled	39,588 (12,810–66,365)	9415 (7192- 11,639)	54,058 (49,567- 58,548)
b. Median OOPE for per episode of hospitalisation (in INR) with 95% CI			
(*N* = 4,616)	Median OOPE (CI)	Public facilities	Private facilities
All	6510 (6050–7000)	2100 (2000–2400)	13,500 (12,200–15,000)
PFHI Enrolled	5450 (4800–6182)	1610 (1256–2010)	13,000 (11,000–15,000)
Not enrolled	7000 (6379–7500)	2300 (2000–2600)	13,530 (12,100–15,000)

The mean OOPE for utilising private hospitals was lower for the PFHI-enrolled individuals than the non-enrolled individuals. However, the median OOPE for hospitalisation in private hospitals was almost equal for the PFHI-enrolled and the non-enrolled individuals.

### Catastrophic health expenditure (CHE)

Overall, the incidence of CHE25 was around four times greater for utilising private hospitals in comparison to the public hospitals. Around 30% of the episodes in private facilities resulted in occurrence of CHE25 when the individuals involved were covered under PFHI. The incidence of CHE25 among those enrolled under PFHI was also about four times greater for hospitalisation in private facilities as compared to public facilities (Table 5). The pattern of CHE25 among those not enrolled under PFHI was also similar.

**Table 5:** Catastrophic Health Expenditure incidence at 25% (CHE-25) and 40% (CHE-40 Non-foods)

PFHI	Type of health facility	CHE-25% with 95% CI ( )	CHE-40% (Non Food expenditure) with 95% CI ( )
Overall	Overall	22.47 (37.3–49.9)	44.1 (40.4–47.8)
	Public	6.9 (5.2–9.1)	22.6 (9.5–26.1)
	Private	31.3 (5.4–37.9)	56.1 (51.6–60.6)
PFHI Enrolled	Overall	19.1 (15.8–22.8)	40.8 (36.1–45.8)
	Public	7.01(5.1–9.7)	17.3 (13–22.8)
	Private	30.01 (26.3–33.9)	59.3 (53.4–64.9)
Not Enrolled	Overall	23.4 (18.2–29.4)	44.9 (40.5–49.5)
	Public	8.6 (7.2–10.2)	24.5 (20.7–28.8)
	Private	28.2 (26.3–30.3)	55.4 (49.9–60.7)

The incidence of CHE40 among the PFHI-enrolled was around three times greater for hospitalisations in private facilities as compared to public facilities (Table 5). More than half of the hospitalisations in private sector resulted in CHE40 irrespective of the PFHI-enrolment status of the individuals.

### Effect of PFHI enrolment on size of OOPE

The quantile regression analysis for OOPE showed that there was no significant association between PFHI-enrolment and size of OOPE. Utilisation of private facilities was associated significantly with greater OOPE as compared to public facilities. Longer duration hospitalisations resulted in greater OOPE (Additional File S1). Hospitalisations of the elderly living in urban areas involved greater OOPE than those from rural areas. Hospitalisation OOPE was likely to be greater for the richer individuals. Hospitalisation OOPE was likely to be greater for the individuals with higher educational attainment. Hospitalisations of men involved greater OOPE than the women. OOPE also varied according to the type of disease and the state of residence. The OLS model also showed a similar pattern of results (Additional File S1).

The PSM model showed that the PFHI-enrolment did not have a significant effect on size of OOPE for hospitalisations of the elderly (Table 6).

**Table 6:** PSM Model: Effect of enrolment under PFHI on OOPE and CHE

Variables		Average Treatment Effect on Treated (ATET) in PSM Model		
		**Coeff**	**95% CI of Coeff**	***p* Value**
**PFHI**	OOPE	511.6	-3547.3 to 4570.6	0.805
	CHE25	0.08	-3.1 to 3.2	0.96
	CHE40	2.7	-0.16 to 5.6	0.06

### Effect of PFHI enrolment on CHE25 and CHE40

The logistic regression model showed that CHE25 was not significantly associated with the PFHI-enrolment status of the hospitalised individuals (OR = 1.01, *p* = 0.92) (Additional File S2). Those using public facilities were significantly less likely to incur CHE25 (OR = 0.17, *p* < 0.01). Longer duration hospitalisations were more likely to result in CHE25. Hospitalisations of men involved greater incidence of CHE25 than women. Hospitalisations of those in the poorest quintile involved greater incidence of CHE25 than those in middle quintile. Incidence of CHE25 varied for different diseases and states (Additional File S2). The logistic regression for CHE40 showed a similar pattern (Additional file S2).

The PSM Model showed no effect of PFHI-enrolment on CHE25 (Table 6). The same finding was there for CHE40 (Table 6).

## Discussion

The elderly are one of the most important parts of the population who need to be protected from large healthcare expenditure if countries are to make progress towards the goal of UHC. In India, PFHI is the key policy to achieve this end. The present study is the first study in India that has examined the effect of PFHI on financial protection of the elderly population. It utilised a large survey of elderly individuals known as the Longitudinal Aging Study (LASI) Wave 1 conducted in 2017–18. The survey provided the large and nationally representative sample required for such an evaluation on a national scale. The present study computed the out of pocket expenditure and catastrophic health expenditure as key measures of financial protection. Since PFHI in India is focused on inpatient care, the study examined the effect of PFHI on financial protection for inpatient care of the elderly.

The current study found that 7.1% of the elderly (aged 45 and above) got hospitalised over a year. According to another study of the same period (2017–18), the hospitalisation rate was 8.5% for the above 60 years age group. The difference may be due to the different age groups in the two studies.

The current study found that 35% of hospitalisations of the elderly (age 45 and above) took place in public facilities. Another study of the same period was based on the National Sample Survey (NSS) dataset and it reported the share of public sector as 40% for the above 60 age group [30].

The current study found that enrolment under PFHI increased the chances of using public facilities for hospitalisation. This is surprising, considering that one of the objectives of PFHI was to expand access to hospital care by making the private sector affordable to the poor. This phenomenon may be related to failure of PFHI in actually improving the affordability of private sector [20, 21, 29, 31, 32].

The key finding of the current study is that enrolment under PFHI was not effective in improving financial protection for the elderly. Though the existing studies in India have not examined effectiveness of PFHI for the elderly, most of them have reported that PFHI could not reduce OOPE or catastrophic expenditure for the overall population [8, 20, 21, 29, 31–33]. Many of the existing studies on PFHI have been based on analysis of National Sample Survey (NSS) dataset on healthcare utilisation. The current study is the first one to use the LASI dataset. This shows that the effectiveness of PFHI has been poor in India, whether examined through NSS or LASI datasets.

Studies from other LLMICs on OOPE incurred by the elderly have also shown a lack of evidence in favour of PFHI. In China, there was some improvement in access to healthcare with PFHI but such schemes could not protect the elderly from large OOPE [19, 34]. In Philippines, a study has shown that enrolment under PFHI resulted in increase in OOPE for the elderly [35]. In Mexico, studies have shown the ineffectiveness of PFHI in reducing OOPE [36, 37].

The current study found that utilisation of private facilities involved substantially greater financial risk as compared to public facilities, irrespective of the PFHI. A similar finding has been reported by a number of Indian studies, though not in the context of the elderly [20, 21, 24]. The private hospitals entered into contracts under PFHI that forbade them from charging anything from patients. However as the current study shows, they continued to charge from patients enrolled under PFHI. Private hospitals in India are poorly regulated [28–30]. Purchasing arrangements through contracts also seem to be ineffective in ensuring price regulation in India’s private sector [20, 21, 24, 31–33, 38, 39]. A question arises why adherence to the contracts could not be ensured. Some have pointed out the 'provider capture' as a possible explanation [40, 41]. The private hospitals in India wield significant economic and political clout to thwart the chances of any punitive action when they flout the contracts [41, 42]. A qualitative study has concluded that the normative and cultural context of private sector provisioning in India is a key constraint [33]. The failure of PFHI could be due to a combination of gaps—of poor government regulation, cultural acceptance of illegal payments and treating healthcare as a market transaction [33].

In the current study, the elderly utilising public facilities faced less incidence of catastrophic expenditure, irrespective of their enrolment under PFHI. A study from Mexico also found the use of public facilities by elderly to have a protective effect [43].

Further research is recommended to understand the governance gaps that lead to poor performance of PFHI in India.

Further research is also recommended on financial protection policies for the elderly in India, including by using the future waves of the LASI survey.

### Limitations

The current study is cross-sectional whereas two rounds of measurement would be ideal for an evaluation. The current study did not have the option of using two rounds as only the first wave of LASI had been completed. The quality of care could not be included as a variable.

## Conclusion

This is the first study in India that examines the performance of PFHI in providing financial protection to the elderly population from hospitalisation costs. Based on the analysis presented in the present study, we conclude that PFHI was not effective in financial protection of the elderly. The ongoing reliance of such policies on a poorly regulated private sector seems to be a big limitation. Further studies are recommended to find out the reasons of poor performance of PFHI for the elderly in LLMIC contexts such as India’s. The goal of UHC cannot be realized unless the elderly are protected from financial risk of healthcare costs. Governments need to find more effective ways of ensuring affordable healthcare for the elderly.

## Supplementary Information

**Additional file 1.** **Table: **OLS and Quantile regression for OOPE.

**Additional file 2.** **Table: **Logistic regression models for CHE25 and CHE40.

## Data Availability

The dataset used and analyzed during the current study is available in public domain. It is freely available for research and academic purposes. It can be requested at https://www.iipsindia.ac.in/content/LASI-data using request form https://iipsindia.ac.in/sites/default/files/LASI_DataRequestForm_0.pdf

## Abbreviations

CHE: Catastrophic Health Expenditure
CHE40: Catastrophic Health Expenditure at threshold of 40% of annual non-food expenditure of household
CHE25: Catastrophic Health Expenditure at threshold of 25% of annual expenditure of household
CI: Confidence Interval
INR: Indian Rupees
LLMICs: Low and Low- to Middle-Income Countries
OOPE: Out-of-pocket expenditure
PFHI: Publicly Funded Health Insurance
