# Supplementary material for: Role of publicly funded health insurance in financial protection of the elderly from hospitalisation expenditure in India-findings from the longitudinal aging study
Source: BMC Geriatr. 2022 Jul 12;22:572. doi: 10.1186/s12877-022-03266-2 (PMC9275032; doi:10.1186/s12877-022-03266-2)
Supplement: Supplementary file 1 — Additional file 1. Table: OLS and Quantile regression for OOPE. [file 12877_2022_3266_MOESM1_ESM.docx]

| **Additional File S1** |  | |  |  |  |  |  | |
| --- | --- | --- | --- | --- | --- | --- | --- | --- |
| **Table: OLS and Quantile regression for OOPE** | | | | | | | | |
| **Out of Pocket Expenditure** | OLS | | 50 Quintile | 20 Quintile | 40 Quintile | 60 Quintile | | 80 Quintile |
|  | Coefficient | | | | | | | |
| **Residence** |  | | | | | | | |
| Rural | 1 | |  | | | | | |
| Urban | 7327** | | 1500* | 411 | 1044* | 1976* | 2645 | |
| **MPCE Quintile** |  | | | | | | | |
| Poorest | 1 | |  | | | | | |
| Poorer | -1230 | | 522 | 459 | 558 | 237 | 601 | |
| Middle | 1201 | | 1522 | 766 | 1114 | 1390 | 1020 | |
| Richer | 5781* | | 2834* | 1128* | 2047* | 3167* | 3279** | |
| Richest | 19103** | | 5546** | 1783** | 3847** | 6927** | 14906** | |
| **Caste (Social Group)** |  | | | | | | | |
| Scheduled Tribe | 1 | |  | | | | | |
| Scheduled Caste (SC) | -820 | | 401 | -61 | 345 | 526 | 692 | |
| Other Backward Class | -862 | | 288 | 206 | 498 | 661 | 880 | |
| None of above | 1546 | | 896 | 381 | 1138 | 1169 | 2383 | |
| **Age** |  | | | | | | | |
| 45-59 Years | 1 | |  | | | | | |
| 60-79 Years | 1328 | | 238 | -263 | -89 | 462 | 595 | |
| 80 and Above | 3097 | | 785 | -333 | 188 | 1087 | 1711 | |
| **Education Category** |  | | | | | | | |
| No Schooling | 1 | |  | | | | | |
| Primary | -1501 | | -196 | -61 | -131 | 50 | 384 | |
| Secondary | 9 | | 1684 | 47 | 718 | 1999 | 2268 | |
| Higher Secondary or Diploma | 3282 | | 4801** | 829 | 3156** | 6862** | 7025 | |
| Graduation and above | 14952** | | 9870** | 470 | 3742** | 11930** | 19666** | |
| **Sex** |  | | | | | | | |
| Male | 1 | |  | | | | | |
| Female | -3698* | | 126 | 131 | 173 | 74 | -1083.698 | |
| **Type of Provider** |  | | | | | | | |
| Public | 1 | |  | | | | | |
| Private | 18779** | | 8535** | 3221** | 6765** | 9928** | 15944** | |
| **Duration of hospitalisation (days)** | 1483** | | 1054** | 236** | 765** | 1415** | 2201** | |
| **PFHI enrolled** | -860 | | 200 | -226 | -490 | -227 | -319 | |
| **Disease** |  | | | | | | | |
| Cancer | 1 |  | | | | | | |
| Chronic pain in joints/arthritis | -21686** | -21038** | | -5232** | -8736** | -21873** | | -34504** |
| Dengue or other vector-born disease | -31722** | -20843** | | -4764** | -9322** | -21893** | | -35985** |
| Depression or anxiety/tension | -26322** | -19247** | | -4562** | -7943** | -21785** | | -36094** |
| Diabetes or related complications | -28285** | -20551** | | -4385** | -8479** | -22031** | | -36381** |
| Fever/Pyrexia of unknown reason | -30392** | -21786** | | -5069** | -9290** | -23447** | | -36724** |
| Fracture/Muscle rupture | -16942* | -15671** | | -3936** | -5558** | -14831** | | -19596** |
| Gastroenteritis | -24845** | -20594** | | -4602** | -8273** | -21482** | | -35221** |
| High blood pressure (hypertension) | -26919** | -20460** | | -4836** | -8474** | -21932** | | -37123.72 |
| HIV/AIDS | -24430 | -21895 | | -3317 | -9181 | -21405 | | -41152 |
| Injury/accident (non-occupational) | -20267** | -18466** | | -4090** | -7314** | -19944** | | -30317** |
| Liver diseases | -22696** | -18601** | | -4556** | -6678** | -19807** | | -34675** |
| Malaria | -27254** | -21073** | | -4614** | -8744** | -22984** | | -38056.6 |
| Maternal or Prenatal Conditions | -15007* | -13742** | | -1192 | -1951 | -11274** | | -24487** |
| Occupation/work-related accident/injury | -24502** | -18997** | | -4342** | -7112** | -20529** | | -26485* |
| Other acute/chronic communicable disease | -23788** | -18624** | | -3919** | -5738* | -19968** | | -33240** |
| Problems with breathing | -23437** | -20309** | | -3862** | -7992** | -21714** | | -34563** |
| Problems with heart | 92.00 | -13830** | | -3397** | -5079** | -12138** | | -3754 |
| Stroke/sudden paralysis of one side | -9895 | -15975** | | -3049** | -5294** | -17651** | | -26160** |
| Surgery for abdominal causes | -17198** | -16431** | | -3030** | -4893* | -17182** | | -25517** |
| Surgery for genitourinary cause | -21681** | -15400** | | -3853** | -4209 | -14837** | | -28561** |
| Surgery for ophthalmic cause | -23811** | -19416** | | -4830** | -7502** | -20127** | | -33420** |
| Surgery for other causes | -10825 | -16721** | | -3660** | -5582* | -13159** | | -25255** |
| Tuberculosis | -22489* | -18988** | | -3734* | -6903* | -20764** | | -33542** |
| Upper Respiratory Tract Infection | -19481 | -17483** | | -4056* | -6147* | -18831** | | -31957** |
| Urinary Tract Infection (UTI) | -17033* | -15966** | | -2639 | -5372* | -17027** | | -25959** |
| Others | -23705* | -19856** | | -5153** | -8064** | -20950** | | -33969** |
| **State** |  | | | | | | | |
| Andhra Pradesh | 1 | |  | | | | | |
| Arunachal Pradesh | 25098** | | 10519** | 4872** | 8202** | 13985** | 22416* | |
| Assam | 14666* | | 8083** | 1849 | 5033** | 9291** | 17084* | |
| Bihar | 13814* | | 1986 | 1794 | 2169 | 3272 | 6318 | |
| Chandigarh | 6269 | | 4238 | 1145 | 3040 | 4904 | 6556 | |
| Chhattisgarh | 8679 | | 2787 | 544 | 2790 | 4031 | 4224 | |
| Dadra and Nagar Haveli | 11241 | | 1726 | 322 | 1637 | 2739 | 4732 | |
| Daman and Diu | 4131 | | 177 | -1025 | -429 | 1960 | 2469 | |
| Delhi | 19414** | | -701 | -1162 | -164 | 530 | 1346 | |
| Goa | 7156 | | 726 | 23 | 44 | 1000 | 1676 | |
| Gujarat | 6415 | | -205 | -819 | -208 | 721 | 3103 | |
| Haryana | 4102 | | -1246 | -269 | -1111 | -952 | 276 | |
| Himachal Pradesh | 12009* | | 3925 | 1615 | 2808 | 3906 | 4078 | |
| Jammu and Kashmir | 7642 | | 2839 | 1544 | 2270 | 3323 | 5454 | |
| Jharkhand | 5073 | | 378 | 1978 | 837 | 1651 | 2904 | |
| Karnataka | 9327 | | 2507 | 702 | 1905 | 5413* | 6685 | |
| Kerala | 5838 | | -153 | 460 | 70 | 533 | 2408 | |
| Lakshadweep | 17769* | | 3717 | 432 | 2194 | 5042 | 16478* | |
| Madhya Pradesh | 4220 | | 1560 | 497 | 1213 | 1892 | 3160 | |
| Maharashtra | 6504 | | 1809 | 816 | 1568 | 2785 | 3662 | |
| Manipur | 15771* | | 4800 | 2640* | 3744* | 6581* | 8156 | |
| Meghalaya | 18622* | | 4075 | 2811 | 4235 | 5032 | 7094 | |
| Mizoram | 7223 | | 4424 | 4437** | 5128* | 5451 | 3879 | |
| Nagaland | 16595 | | 11445** | 1907 | 8017** | 19273** | 27647** | |
| Odisha | 15780* | | 4788* | 1484 | 3602* | 5017 | 5501 | |
| Puducherry | -2311 | | -1856 | -1049 | -2450 | -885 | -234 | |
| Punjab | 9484 | | 2650 | 1932 | 2163 | 1755 | -287 | |
| Rajasthan | 5698 | | 2353 | 1084 | 2320 | 3295 | 6255 | |
| Tamil Nadu | 8656 | | 1463 | 228 | 1664 | 2525 | 5150 | |
| Telangana | 10108* | | 1469 | -1 | 885 | 2385 | 2879 | |
| Tripura | 14317* | | 3711 | 1737 | 3096 | 4645 | 5106 | |
| Uttar Pradesh | 3273 | | -84 | 346 | -219 | 1354 | 3416 | |
| Uttarakhand | 18248** | | 4681 | 1891 | 4500* | 6444* | 13873 | |
| West Bengal | 2844 | | 1819 | 196 | 1315 | 2358 | 2922 | |
| Andaman and Nicobar | 4762 | | -1446 | -172 | -1573 | -1844 | 366 | |
|  |  | |  |  |  |  |  | |
| * is for p value <0.05 |  | |  |  |  |  |  | |
| ** is for p value < 0.01 |  | |  |  |  |  |  | |
