# Supplementary material for: Role of publicly funded health insurance in financial protection of the elderly from hospitalisation expenditure in India-findings from the longitudinal aging study
Source: BMC Geriatr. 2022 Jul 12;22:572. doi: 10.1186/s12877-022-03266-2 (PMC9275032; doi:10.1186/s12877-022-03266-2)
Supplement: Supplementary file 2 — Additional file 2. Table: Logistic regression models for CHE25 and CHE40. [file 12877_2022_3266_MOESM2_ESM.docx]

| **Additional File S2** |  |  |  |  |  |  |  |  |
| --- | --- | --- | --- | --- | --- | --- | --- | --- |
| **Table: Logistic regression models for CHE25 and CHE40** | | | | | | | | |
|  | **CHE25** | | | | **CHE40** | | | |
|  | **Odds** | **[95% CI]** | | **p Value** | **Odds** | **[95% CI]** | | **p Value** |
| **Residence** |  | | | | | | | |
| Rural | 1 |  | | | | | | |
| Urban | 0.99 | 0.80 | 1.22 | 0.90 | 0.97 | 0.76 | 1.24 | 0.80 |
| **MPCE quintile** |  | | | | | | | |
| Poorest | 1 |  | | | | | | |
| Poorer | 0.82 | 0.56 | 1.19 | 0.30 | 0.67 | 0.42 | 1.06 | 0.09 |
| Middle | 0.66 | 0.46 | 0.96 | 0.03 | 0.67 | 0.44 | 1.04 | 0.08 |
| Richer | 0.99 | 0.70 | 1.39 | 0.95 | 0.93 | 0.62 | 1.40 | 0.74 |
| Richest | 1.65 | 1.19 | 2.29 | <0.01 | 1.49 | 1.02 | 2.18 | 0.04 |
| **Caste** |  | | | | | | | |
| Scheduled Tribe (ST) | 1 |  | | | | | | |
| Scheduled Caste (SC) | 1.00 | 0.64 | 1.54 | 0.98 | 1.24 | 0.71 | 2.14 | 0.45 |
| Other backward class | 1.15 | 0.77 | 1.71 | 0.50 | 1.55 | 0.94 | 2.56 | 0.09 |
| None of above | 1.14 | 0.76 | 1.72 | 0.53 | 1.67 | 1.00 | 2.79 | 0.05 |
| **Age Category** |  | | | | | | | |
| 45-59 Years | 1 |  | | | | | | |
| 60-79 Years | 1.23 | 1.02 | 1.49 | 0.04 | 1.25 | 1.00 | 1.56 | 0.05 |
| 80 and Above | 1.07 | 0.71 | 1.62 | 0.75 | 1.10 | 0.68 | 1.78 | 0.69 |
| **Education Category** |  | | | | | | | |
| No Schooling | 1 |  | | | | | | |
| Primary | 0.83 | 0.65 | 1.05 | 0.12 | 0.77 | 0.58 | 1.03 | 0.08 |
| Secondary | 1.00 | 0.77 | 1.31 | 1.00 | 0.86 | 0.63 | 1.18 | 0.35 |
| Higher Secondary and diploma | 1.10 | 0.73 | 1.67 | 0.64 | 0.88 | 0.55 | 1.43 | 0.61 |
| Graduation and above | 1.39 | 0.90 | 2.14 | 0.14 | 1.21 | 0.75 | 1.98 | 0.43 |
| **Sex** |  | | | | | | | |
| Male | 1 |  | | | | | | |
| Female | 0.74 | 0.61 | 0.90 | <0.01 | 0.68 | 0.54 | 0.85 | <0.01 |
| **Use of Public Facility** | 0.17 | 0.13 | 0.21 | <0.01 | 0.17 | 0.12 | 0.22 | <0.01 |
| **Duration of hospitalisation** | 1.09 | 1.08 | 1.11 | <0.01 | 1.07 | 1.06 | 1.09 | <0.01 |
| **PFHI-enrollment** | 1.01 | 0.80 | 1.28 | 0.92 | 1.09 | 0.83 | 1.42 | 0.54 |
| **Disease** |  | | | | | | | |
| Cancer | 1 |  | | | | | | |
| Chronic pain | 0.19 | 0.10 | 0.36 | <0.01 | 0.20 | 0.10 | 0.38 | <0.01 |
| Dengue | 0.09 | 0.03 | 0.23 | <0.01 | 0.07 | 0.02 | 0.21 | <0.01 |
| Depression | 0.20 | 0.07 | 0.58 | <0.01 | 0.27 | 0.09 | 0.81 | 0.02 |
| Diabetes | 0.17 | 0.08 | 0.34 | <0.01 | 0.11 | 0.05 | 0.24 | <0.01 |
| Fever/Pyrexia | 0.09 | 0.05 | 0.18 | <0.01 | 0.02 | 0.01 | 0.07 | <0.01 |
| Fracture/Muscle | 0.47 | 0.24 | 0.90 | 0.02 | 0.35 | 0.18 | 0.69 | <0.01 |
| Gastroenteritis | 0.17 | 0.09 | 0.34 | <0.01 | 0.14 | 0.07 | 0.30 | <0.01 |
| High blood pressure | 0.13 | 0.06 | 0.27 | <0.01 | 0.10 | 0.05 | 0.23 | <0.01 |
| HIV/AIDS | 1.00 |  |  |  | 1.00 |  |  |  |
| Injury/accident | 0.37 | 0.19 | 0.72 | <0.01 | 0.32 | 0.16 | 0.64 | <0.01 |
| Liver diseases | 0.42 | 0.20 | 0.88 | 0.022 | 0.33 | 0.15 | 0.73 | <0.01 |
| Malaria | 0.12 | 0.04 | 0.31 | <0.01 | 0.12 | 0.04 | 0.34 | <0.01 |
| Maternal or Pren | 1.01 | 0.49 | 2.07 | 0.99 | 0.72 | 0.34 | 1.55 | 0.40 |
| Occupation/work | 0.30 | 0.12 | 0.77 | 0.01 | 0.27 | 0.10 | 0.73 | <0.01 |
| Other acute illness | 0.33 | 0.14 | 0.77 | 0.01 | 0.21 | 0.08 | 0.56 | <0.01 |
| Problems with Breathing | 0.23 | 0.11 | 0.45 | <0.01 | 0.17 | 0.08 | 0.36 | <0.01 |
| Problems with Heart | 0.62 | 0.33 | 1.14 | 0.12 | 0.51 | 0.27 | 0.95 | 0.04 |
| Stroke | 0.48 | 0.25 | 0.94 | 0.03 | 0.32 | 0.16 | 0.64 | <0.01 |
| Surgery for abdominal causes | 0.42 | 0.23 | 0.78 | <0.01 | 0.29 | 0.15 | 0.56 | <0.01 |
| Surgery for genitourinary | 0.26 | 0.12 | 0.57 | <0.01 | 0.21 | 0.09 | 0.50 | <0.01 |
| Surgery for ophthalmic cause | 0.28 | 0.14 | 0.56 | <0.01 | 0.15 | 0.07 | 0.34 | <0.01 |
| Surgery for other causes | 0.64 | 0.32 | 1.32 | 0.23 | 0.52 | 0.25 | 1.09 | 0.09 |
| Tuberculosis | 0.20 | 0.06 | 0.72 | 0.01 | 0.13 | 0.03 | 0.63 | <0.01 |
| Upper Respiratory Tract Infection | 0.63 | 0.23 | 1.76 | 0.38 | 0.16 | 0.04 | 0.64 | <0.01 |
| Urinary Tract Infection | 0.45 | 0.20 | 1.00 | 0.05 | 0.45 | 0.19 | 1.02 | 0.06 |
| Others | 0.22 | 0.09 | 0.50 | <0.01 | 0.16 | 0.06 | 0.42 | <0.01 |
| **State** |  | | | | | | | |
| Andhra Pradesh |  |  |  |  |  |  |  |  |
| Arunachal Pradesh | 13.60 | 5.89 | 31.42 | <0.01 | 10.37 | 3.92 | 27.4 | <0.01 |
| Assam | 3.85 | 1.92 | 7.74 | <0.01 | 1.49 | 0.62 | 3.57 | 0.37 |
| Bihar | 1.64 | 0.90 | 2.99 | 0.11 | 1.33 | 0.67 | 2.65 | 0.41 |
| Chandigarh | 1.19 | 0.44 | 3.21 | 0.74 | 1.09 | 0.34 | 3.48 | 0.88 |
| Chhatisgarh | 1.06 | 0.39 | 2.88 | 0.91 | 0.76 | 0.22 | 2.57 | 0.65 |
| Dadra and Nagar Haveli | 2.20 | 0.94 | 5.15 | 0.07 | 2.05 | 0.76 | 5.50 | 0.15 |
| Daman and Diu | 0.99 | 0.40 | 2.47 | 0.98 | 1.12 | 0.40 | 3.14 | 0.83 |
| Delhi | 1.11 | 0.43 | 2.82 | 0.83 | 1.21 | 0.43 | 3.43 | 0.72 |
| Goa | 1.09 | 0.52 | 2.31 | 0.82 | 1.30 | 0.57 | 2.95 | 0.54 |
| Gujarat | 0.83 | 0.43 | 1.62 | 0.59 | 0.53 | 0.23 | 1.20 | 0.13 |
| Haryana | 1.06 | 0.54 | 2.08 | 0.86 | 0.88 | 0.40 | 1.94 | 0.74 |
| Himachal Pradesh | 1.80 | 0.91 | 3.56 | 0.09 | 1.03 | 0.45 | 2.37 | 0.94 |
| Jammu and Kashmir | 1.75 | 0.68 | 4.55 | 0.25 | 0.36 | 0.07 | 1.76 | 0.21 |
| Jharkhand | 1.70 | 0.89 | 3.25 | 0.11 | 1.65 | 0.80 | 3.41 | 0.18 |
| Karnataka | 2.27 | 1.28 | 4.04 | <0.01 | 1.48 | 0.76 | 2.87 | 0.25 |
| Kerala | 1.15 | 0.63 | 2.10 | 0.65 | 1.20 | 0.61 | 2.36 | 0.59 |
| Lakshadweep | 2.98 | 1.23 | 7.21 | 0.02 | 4.04 | 1.51 | 10.82 | 0.01 |
| Madhya Pradesh | 0.85 | 0.46 | 1.57 | 0.60 | 0.65 | 0.31 | 1.35 | 0.25 |
| Maharashtra | 2.10 | 1.26 | 3.50 | 0.01 | 1.33 | 0.73 | 2.40 | 0.35 |
| Manipur | 2.07 | 0.99 | 4.32 | 0.05 | 1.52 | 0.65 | 3.54 | 0.33 |
| Meghalaya | 1.80 | 0.62 | 5.24 | 0.28 | 1.30 | 0.34 | 4.99 | 0.70 |
| Mizoram | 1.96 | 0.78 | 4.94 | 0.15 | 1.97 | 0.65 | 5.99 | 0.23 |
| Nagaland | 4.38 | 1.63 | 11.81 | <0.01 | 3.73 | 1.18 | 11.75 | 0.03 |
| Odisha | 2.29 | 1.19 | 4.42 | <0.01 | 1.90 | 0.90 | 4.01 | 0.09 |
| Puducherry | 0.83 | 0.35 | 1.93 | 0.66 | 0.87 | 0.34 | 2.24 | 0.78 |
| Punjab | 0.67 | 0.34 | 1.32 | 0.25 | 0.60 | 0.27 | 1.32 | 0.20 |
| Rajasthan | 1.38 | 0.72 | 2.61 | 0.33 | 1.34 | 0.66 | 2.76 | 0.42 |
| Tamil Nadu | 2.37 | 1.33 | 4.23 | <0.01 | 1.97 | 1.02 | 3.80 | 0.04 |
| Telangana | 1.44 | 0.82 | 2.52 | 0.21 | 1.48 | 0.79 | 2.76 | 0.22 |
| Tripura | 1.10 | 0.45 | 2.73 | 0.83 | 1.37 | 0.50 | 3.75 | 0.54 |
| Uttar Pradesh | 1.56 | 0.89 | 2.75 | 0.12 | 1.61 | 0.85 | 3.02 | 0.14 |
| Uttarakhand | 2.76 | 1.36 | 5.59 | 0.01 | 1.84 | 0.82 | 4.14 | 0.14 |
| West Bengal | 1.32 | 0.71 | 2.46 | 0.38 | 0.87 | 0.41 | 1.84 | 0.72 |
| Andaman Nicobar | 1.12 | 0.39 | 3.19 | 0.84 | 0.77 | 0.20 | 2.94 | 0.70 |
